# Service delivery challenges in HIV care during the first year of the COVID‐19 pandemic: results from a site assessment survey across the global IeDEA consortium

**DOI:** 10.1002/jia2.26036

**Published:** 2022-12-11

**Authors:** Ellen Brazier, Rogers Ajeh, Fernanda Maruri, Beverly Musick, Aimee Freeman, C. William Wester, Man‐Po Lee, Tinei Shamu, Brenda Crabtree Ramírez, Marcelline d'Almeida, Kara Wools‐Kaloustian, N. Kumarasamy, Keri N. Althoff, Christella Twizere, Beatriz Grinsztejn, Frank Tanser, Eugène Messou, Helen Byakwaga, Stephany N. Duda, Denis Nash, the International epidemiology Databases to Evaluate AIDS, Chidchon Chansilpa, Trevor Dougherty, Azar Karminia, Matthew Law, Jeremy Ross, Annette Sohn, Ivette Aguirre, David Baker, Mark Bloch, Safaa Cabot, Andrew Carr, Deborah Couldwell, Sian Edwards, Beng Eu, Heather Farlow, Robert Finlayson, Manoji Gunathilake, Cherie Hazlewood, Jennifer Hoy, Julian Langton‐Lockton, Jacqueline Le, Elizabeth Leprince, Ariane Minc, Richard Moore, Maree O'Sullivan, Norm Roth, Dianne Rowling, Darren Russell, Nathan Ryder, Craig Saunders, Julie Silvers, David J. Smith, David Sowden, Grant Sweeney, Lynn Tan, Ricard Teague, David Templeton, Caroline Thng, Ian Woolley, Vohith Khol, Penh Sun Ly, Tsz Hei Li, Lee Man Po, Aarti Kinikar, Nagalingeswaran Kumarasamy, Sanjay Mundhe, Sanjay Pujari, Shashikala Sangle, Smita Nimkar, Madelein Jassin, Nia Kurniati, Tuti Parwati Merati, Dina Muktiarti, Rizqi Amalia, Ni Made Dewi Dian Sukmawati, Ketut Dewi Kumara Wati, Evy Yunihastuti, Junko Tanuma, Jun Yong Choi, Raja Iskandar Shah Raja Azwa, Chan Kwai Cheng, Yasmin Mohamed Gani, Thahira Jamal Mohamed, Fong Siew Moy, Revathy Nallusamy, Mohamad Zulfahami Mohd Nor, Nuraini Rudi, Wong Peng Shyan, Nik Khairulddin Nik Yusoff, Rossana Ditangco, Yu‐Jiun Chan, Pei‐Chieh Wu, Ping‐Feng Wu, Anchalee Avihingsanon, Romanee Chaiwarith, Kulkanya Chokephaibulkit, Suwimon Khusuwan, Sasisopin Kiertiburanakul, Pope Kosalaraksa, Pagakrong Lumbiganon, Pradtana Ounchanam, Thanyawee Puthanakit, Supattra Rungmaitree, Nuttarika Solai, Tavitiya Sudjaritruk, Vu Thien An, Do Duy Cuong, Chau Viet Do, Bui Vu Huy, Tuan Quy, Kinh Van Nguyen, Luan Nguyen, Van Lam Nguyen, Yen Thi Nguyen, Vuong Minh Nong, Huu Khanh Truong, Ngo Thi Thu Tuyen, Catherine C. McGowan, Stephany Duda, Fernanda Maruri, C. William Wester, Florencia Cahn, Pedro Cahn, Carina Cesar, Valeria Fink, Omar Sued, Lara Coelho, Daisy Maria Machado, Jorge Pinto, Marcelo Wolff, Vanessa Rouzier, Denis Padgett, Brenda Crabtree Ramírez, Eduardo Gotuzzo, Ellen Brazier, Denis Nash, Jérémie Biziragusenyuka, Patrick Gateretse, Pelagie Nimbona, Olive Niyonkuru, Christelle Twizere, Rogers Ajeh, Surreng Anicetus, Amadou Djenabou, Priscilla Enow, Eyongetah Mbu, Martin Manga, Mercy Ndobe, Judith Nasah, Elle Nathalie Syntyche Ekossono, Mireille Teno Bouseko, Faustin Kitetele, Patricia Lelo, Merlin Isidore Justin Diafouka, Adolphe Mafoua, Dominique Mahambou Nsonde, Uitonze Aime Maurice Bihira, Marie Chantal Dusabe, Rosine Feza, Jean Claude Habanabashaka, Viateur Habumuremyi, Ernestine Igizeneza, Anne Marie Kamigisha, Gallican Kubwimana, Gilbert Maniriho, Gilbert Mbaraga, Benjamin Muhoza, Jeanne Mukakarangwa, Joyce Mukamana, Patricie Mukanyirigira, Yvone Claude Mukeshimana, Athanase Munyaneza, Gad Murenzi, Jacqueline Musaninyange, Jules Ndumuhire Nyiraneza, Fidele Ntarambirwa, Marie Louise Nyiraneza, Josette Tuyishime, Yvonne Tuyishimire, Alexis Ubandutira, Florance Umugiraneza, Rosine Umugwaneza, Olive Uwamahoro, Pauline Uwamahoro, Marie Victoire Uwambaje, Clarisse Uwimpuhwe, Siphora Uwiragiye, Yee Yee Kuhn, Beverly Musick, Kara Wools‐Kaloustian, Felix Adera, Beatricec Adhiambo, Khaemba Aggrey, Daniel Akadikor, Felix Ambulla, Dorah Apiyo, Patrick Ariya, Naftal Atemba, Fridah Ayodi, Chirchir Benard, Maureen Bett, Serafine Birgen, Rael Bwalei, Nancy Chebon, Valentine Jirry Chebor, Philip Chebuiywo, Jacline Chemutai, Emily Chepkorir, Carolyne Chepseba, John Chirchir, John Chirchir, Lameck Diero, Benard Dukwa, Alice Elphas, Tom Etyang, Agnes Idiama, Ann Jebichuko, Delvine Jepchumba, Churchill Juma, Maureen Juma, Sheila Juma, Julie Kadima, Rose Karani, Christopher Keitany, Pricilla Keter, Lucy Kiavoga, Harrison Kibet, Ruth Kimutai, Mutai Kiplagat, Wilfred Kiprono, Nicholas Kogei Kipruto, Asenath Kirimi, Zeddy Koech, Carolyne Kosgei, Karen Kutto, Mildred Kweyu, Ephraim Kenneth Liech, Milka Limo, Rose Maina, Priscah Marumbu, Agnes Masese, Patricia Mochotto, Omudeck Molly, Tom Momanyi, John W. Murutu, Praxidis Mwanda, Lillian Ndakalu, Rose N. Nderitu, Sarah Obatsa, Fredrick Obiga, Moses Oboya, Joseph Odhiambo, George Olaya, Oscar Omanyala, Christine Oray, Molly Otieno, Modesta Toto Otwane, Paul Ouma, Charles Owuor, Doris Tutu Pepela, Collins Pessah, Evans Rotich, Edwin K. Rotich, Titus C. Rutto, Monica Shikuku, Rose Naliaka Sibweche, Robert Wanyonyi Simiyu, Hellen Siria, Michael Some, Winnie Cherotich Songok, Immaculate Tanui, Grace Wafula, Rebecca Wambura, Ellah Wanjala, Carolyne Wanyama, Hellen Wanyonyi, Emmanuel Woyakapel, Wandera Zelbabel, Judy, Kiprop, Beatrice, Leah, Dominic, Tallam, Dikengela Gwimo, Ester Kinyota, Jerome Lwali, Rita Lyamuya, Richard Machemba, Julia Mathias, Lilian Mkombachepa, Athuman Mokiwa, Ombeni Mushi, Charles Ndunguru, Kapella Ngonyani, Charles Nyaga, Happiness Ruta, Mark Urassa, James Akanyihayo, Arnold Arinaitwe, Jesca Batuuka, Walusimbi Birungi, John Nyanzi Bugembe, Ahmed Ddungu, Kato Francis, Bangira Imran, George William Kafuuma, John Bosco Kalulue, Grace Kanaabi, Michale Kanyesigye, Godfery Karuhanga, Charles Kasozi, Godfrey Kasule, Assumpta Katusime, Donozio Kibalama, Donozio Kibalama, Simon Peter Kimera, Namatovu Kulusumu, Yusuf Lule, Isaac Lwanga, Margaret Mluindwa, Jemba Moses, Sseremba Mubarak, Daniel Muggaga, Evelyn Mukalazi, Joseph Muleebwa, Derick Mulema, Ivan Musisi, John Muwawu, Winnie Muyindike, Dick Mwaka, Milly Naava, Immaculate Nabiyki, Agnes Nabusulwa, Dorah Nakabugo, Esther Nakamya, Daisy Nakanwagi, Oliver Nakato, Lydian Nakayi, Patience Nakigozi, Juliet Nakku, Juliet Nakuya, Justine Nakyomu, Joan Namayanja, Sarah Namirembe, Juliet Namugumya, Ezereth Namukasa, Viola Namulindwa, Irene Nankya, Grace Mugagga Nannyondo, Harriet Nansamba, Denis Nansera, Brenda Nanyanzi, Esther Celina Nanyonjo, Irene Nayiga, Isaac Opira, Noela C. Owarwo, Sserunkuma Resty, Haruna Semuwemba, Julius Senoga, Gerald Sseguya, John Paul Ssekyewa, Matthew Ssemakadde, Jonah Tebajjwa, Doreen Tugumisirize, Robinah Tushemerirwe, Kawuki Waliyi, Fenehance, Medard, Richard Moore, Keri Althoff, Aimee Freeman, Jennifer Bishop, M J Gill, Mona Loutfy, Graham Smith, Laura Bamford, Anthony Black, Asia Brice, Sheldon Brown, Jonathan Colasanti, Piper Duarte, Cynthia Firnhaber, Matthew Goetz, Chris Grasso, Barbara Gripshover, Michael Horberg, Rita Kelly, Ken Levine, Mitchell Luu, Vincent Marconi, Karen Maroney, Kenneth Mayer, Angel Mayor, Catherine McGowan, Richard Moore, Ami Multani, Sonia Napravnik, Ank Nijhawan, Richard Novak, Frank Palella, Maria C. Rodriguez, Mia Scott, Ellen Tedaldi, James Willig, Morna Cornell, Mary‐Ann Davies, Matthias Egger, Andreas Haas, Monkoe Bereng, Maleshoane Kalake, Keketso Lenela, Relebohile Seretse, Matthews Chintenga, Jane Chiwoko, Joe Gumulira, Jacqueline Huwa, Rafique Maluwa, Beatrice Matanje, Ronald Mbewe, Sunshine Mfungwe, Zakaliah Mphande, Hannock Tweya, Idiovino Rafael, Patti Apolles, Eunice Beneke, Siphephelo Dlamini, Claire Edson, Brian Eley, Jonathan Euvrard, Geoffrey Fatti, Bridgette Goeieman, Ashraf Grimwood, David Huang, Susan Hugo, Zahiera Ismail, Lauren Jennings, Thulile Mathenjwa, Lizette Monteith, Zamuxolo Mshweshwe, Mfundi Ntuli, EN Ndlovu, Hloniphile Ndlozi, Sylvia Noyakaza, Hans Prozesky, Helena Rabie, Nosisa Sipambo, Karl‐Günter Technau, Thokozani Tembe, Nontando Xaba, Thandiwe Njobvu, Mary Munthaly, Elly Mwetwa, Gillian Kabeba, Derrick Mwendafilumba, Ethel Maanguka, Nelly Manyika, Chalwe Mwansa, Future Banda, Dickson Mwenda, Abel Bwalya, Leah Shapi, Kasapo Syame, Rita Sashi, Chisha Mulenga, Ruth Nanyangwe, Cleophas Chimbetete, A. Chinofunga, J. Mhike, E. Mubvigwi, F. Nyika, Kumbirai Pise Quarter, Shino Chassagne Arikawa, Renaud Becquet, Charlotte Bernard, François Dabis, Sophie Desmonde, Désiré Dahourou, Didier Koumavi Ekouevi, Antoine Jaquet, Julie Jesson, Valeriane Leroy, Karen Malateste, Elodie Rabourdin, Thierry Tiendrebeogo, Michée Assogba, Marcelline d'Almeida, Djimon Marcel Zannou, Ghislaine Hounhoui, Denise Bere, Armel Poda, Gbolo Pooda, Richard Traore, Yao Abauble, Ouattara Abby, Patrick Acquah, Valérie Andoble, Yobo N'Dzama Aude, Jean‐Claude Azani, Oka Berete, Jacques Daple Beugre, Caroline Yao Bohoussou, Simon Boni Emmanuel Brou, Henri Chenal, Abdoulaye Cissé, Nambate Coulibaly, Marie Evelyne Dainguy, Marcelle Daligou, Toni Thomas d'Aquin, Claude Desire Dasse, Madeleine Amorissani Folquet, Guy Gnepa, Olivier Gobe, Salif Guira, Denise Hawerlander, Apollinaire Horo, Guillaume Kanga, Zobo Konan Eugène Messou, Kla Albert Minga, Raoul Moh, MarieSylvie N'Gbeche, Patricia Ogbo, Mathieu Oulai, SE Stéphanie, Tanoh Eboua, Itchy Max Valère, Adwoa Kumiwa Asare Afrane, Esther Akrofi, John Christian Andoh, Lorna Renner, Awa Bagayoko, Kadidiatou Bagayoko, Abdou Salam Bah, Alima Berthe, Boureïma Coulibaly, Fatimata Coulibaly, Yacouba Aba Coulibaly, Aïssata Diakité, Fatoumata Bocoum, Fatoumata Boré, Fatoumata Dicko, Odile Koné, Mariam Sylla, Assitan Tangara, Mamadou Traoré, Moussa Seydi, Edmond Amegatse, Julienne Djossou, Elom Takassi, Sénam Palanga

**Affiliations:** ^1^ Institute for Implementation Science in Population Health City University of New York New York New York USA; ^2^ Graduate School of Public Health and Health Policy City University of New York New York New York USA; ^3^ Clinical Research Education Networking and Consultancy Yaoundé Cameroon; ^4^ Department of Medicine, Division of Infectious Diseases Vanderbilt University Medical Center Nashville Tennessee USA; ^5^ Department of Biostatistics and Health Data Science Indiana University School of Medicine Indianapolis Indiana USA; ^6^ Department of Epidemiology Bloomberg School of Public Health Johns Hopkins University Baltimore Maryland USA; ^7^ Queen Elizabeth Hospital Hong Kong China; ^8^ Newlands Clinic Harare Zimbabwe; ^9^ Institute of Social and Preventive Medicine (ISPM) University of Bern Bern Switzerland; ^10^ Departamento de Infectología Instituto Nacional de Ciencias Médicas y Nutrición Mexico City Mexico; ^11^ Centre National Hospitalier, Universitaire Hubert K. Maga Cotonou Benin; ^12^ VHS Infectious Diseases Medical Centre Voluntary Health Services Chennai India; ^13^ Centre National de Reference en Matière de VIH/SIDA Bujumbura Burundi; ^14^ Laboratory of Clinical Research in STD/AIDS (LAPCLIN‐AIDS) Evandro Chagas National Institute of Infectious Diseases‐Oswaldo Cruz Foundation (INI/FIOCRUZ) Rio de Janeiro Brazil; ^15^ Africa Health Research Institute University of KwaZulu‐Natal Durban South Africa; ^16^ ACONDA ‐ Centre de Prise en Charge, de Recherche et de Formation (CePReF) Abidjan Côte d'Ivoire; ^17^ Mbarara University of Science and Technology Mbarara Uganda; ^18^ Department of Biomedical Informatics Vanderbilt University Medical Center (VUMC) Nashville Tennessee USA; ^19^ Vanderbilt Institute for Clinical and Translational Research Vanderbilt University Medical Center (VUMC) Nashville Tennessee USA

**Keywords:** continuity of patient care, COVID‐19, health systems, HIV continuum of care, human immunodeficiency virus, telemedicine

## Abstract

**Introduction:**

Interruptions in treatment pose risks for people with HIV (PWH) and threaten progress in ending the HIV epidemic; however, the COVID‐19 pandemic's impact on HIV service delivery across diverse settings is not broadly documented.

**Methods:**

From September 2020 to March 2021, the International epidemiology Databases to Evaluate AIDS (IeDEA) research consortium surveyed 238 HIV care sites across seven geographic regions to document constraints in HIV service delivery during the first year of the pandemic and strategies for ensuring care continuity for PWH. Descriptive statistics were stratified by national HIV prevalence (<1%, 1–4.9% and ≥5%) and country income levels.

**Results:**

Questions about pandemic‐related consequences for HIV care were completed by 225 (95%) sites in 42 countries with low (*n* = 82), medium (*n* = 86) and high (*n* = 57) HIV prevalence, including low‐ (*n* = 57), lower‐middle (*n* = 79), upper‐middle (*n* = 39) and high‐ (*n* = 50) income countries. Most sites reported being subject to pandemic‐related restrictions on travel, service provision or other operations (75%), and experiencing negative impacts (76%) on clinic operations, including decreased hours/days, reduced provider availability, clinic reconfiguration for COVID‐19 services, record‐keeping interruptions and suspension of partner support. Almost all sites in low‐prevalence and high‐income countries reported increased use of telemedicine (85% and 100%, respectively), compared with less than half of sites in high‐prevalence and lower‐income settings. Few sites in high‐prevalence settings (2%) reported suspending antiretroviral therapy (ART) clinic services, and many reported adopting mitigation strategies to support adherence, including multi‐month dispensing of ART (95%) and designating community ART pick‐up points (44%). While few sites (5%) reported stockouts of first‐line ART regimens, 10–11% reported stockouts of second‐ and third‐line regimens, respectively, primarily in high‐prevalence and lower‐income settings. Interruptions in HIV viral load (VL) testing included suspension of testing (22%), longer turnaround times (41%) and supply/reagent stockouts (22%), but did not differ across settings.

**Conclusions:**

While many sites in high HIV prevalence settings and lower‐income countries reported introducing or expanding measures to support treatment adherence and continuity of care, the COVID‐19 pandemic resulted in disruptions to VL testing and ART supply chains that may negatively affect the quality of HIV care in these settings.

## 1 INTRODUCTION

The COVID‐19 pandemic has had major direct and indirect impacts on population health globally, through disruptions in the accessibility and quality of basic health services [1], in supply chains for essential medications and commodities [2, 3], and in the availability of health workers [4, 5, 6]. These disruptions threaten to slow or reverse progress towards various global health priorities, including efforts to end the HIV epidemic [7, 8]. A modelling group convened by the World Health Organization (WHO) and the Joint United Nations Programme on HIV/AIDS (UNAIDS) in mid‐2020 estimated that a 6‐month disruption of antiretroviral therapy (ART) could lead to close to 500,000 excess deaths from AIDS‐related illnesses, including tuberculosis, in sub‐Saharan Africa in 2020–2021 [9, 10]. Subsequent modelling studies estimated that pandemic‐related disruptions in care could raise new HIV infections and AIDS‐related mortality by 10% over 2–5 years [11, 12], with higher increases among infants and children [13].

In view of the population health impacts of service delivery disruptions for people with HIV (PWH) and those at risk for HIV, the WHO, UNAIDS, the United States President's Emergency Plan for AIDS Relief (PEPFAR) and other partners recommended changes in HIV service delivery to minimize unnecessary clinic visits and risks of exposure to SARS‐CoV‐2 and reduce burdens on healthcare systems, while averting treatment interruption and disengagement from care [14, 15, 16, 17, 18, 19]. Recommended measures included rapid initiation of ART among those not on treatment; expansion of differentiated service delivery (DSD) strategies, such as community distribution of ART and provision of multi‐month supplies of ART, pre‐exposure prophylaxis (PrEP) and tuberculosis preventive therapy; alignment of HIV care with treatment for coinfections and comorbidities; and introduction of telemedicine and virtual consultations.

While there have been efforts to document the impact of the COVID‐19 pandemic on HIV care and treatment programmes, most existing studies have been narrow in geographic, programmatic and temporal scope—that is focused on single clinics [20, 21, 22] or countries [23, 24, 25], as well as services for special populations [26, 27, 28] and the initial months of the pandemic [24, 25, 29]. There are limited cross‐country data on the pandemic's impacts on HIV service delivery, apart from reporting on the expansion of multi‐month dispensing of ART in PEPFAR countries [30], along with disruptions to HIV programmes reported by the Global Fund [31].

This study aimed to document service delivery constraints posed by the COVID‐19 pandemic for HIV care and treatment programmes across diverse country settings, along with strategies used to minimize treatment interruption and care disengagement. Such data are important for understanding and mitigating the impacts of the ongoing pandemic on global efforts to end the HIV epidemic.

## 2 METHODS

### 2.1 Data sources

The International epidemiology Databases to Evaluate AIDS (IeDEA) is a global research consortium comprising HIV care and treatment sites in 44 countries across seven geographic regions: the Asia‐Pacific; the Caribbean, Central and South America (CCASAnet); Central Africa; East Africa; Southern Africa; West Africa; and North America (NA‐ACCORD) [32]. IeDEA regularly conducts general and specialized surveys of participating clinics to collect data on site characteristics and topics related to HIV care that constitute gaps in the scientific literature.

IeDEA's 2020 site assessment survey was a cross‐sectional survey of 238 HIV care and treatment clinics at academic and community‐based hospitals and health centres participating in the consortium in 2020 as described elsewhere [33]. The survey collected data on site characteristics, such as facility type and location, patient population served and routine HIV care (e.g. ART initiation and viral load [VL] monitoring practices) prior to the start of the pandemic. In addition, the survey captured data on the impact of the COVID‐19 pandemic on HIV care via questions exploring whether each site's location (i.e. municipality, district, etc.) had ever been subject to restrictions on travel, service provision or business operations; whether HIV services had ever been suspended because of the pandemic; and the timing and duration of any lockdowns and service‐delivery suspensions. Other questions explored pandemic‐related changes in clinic operations (e.g. staffing shortages, space reconfiguration, use of telemedicine, etc.); community‐based services and programmes for PWH (e.g. HIV testing, support groups, community‐based ART distribution and community tracing); ART initiation and routine ART services; HIV VL testing services; and stockouts of HIV‐related commodities and supplies (e.g. HIV test kits, antiretroviral medications and VL testing supplies). Sites that did not provide a given service prior to the pandemic (e.g. same‐day ART initiation or third‐line ART) were instructed to report “Not applicable” for the service.

The survey was designated a non‐human subjects operational/quality improvement project by the Vanderbilt University Medical Center (VUMC) Institutional Review Board (#200013). Informed consent was not required because the survey collected only site‐level data and did not involve human subjects. Launched in English (11 September 2020) and French (16 October 2020) depending on the country context, the survey was distributed as a self‐administered printable form for completion on paper and as an online questionnaire using REDCap (Research Electronic Data Capture) tools hosted at VUMC [34]. The survey was closed on 1 March 2021.

Site‐level data were linked to national HIV prevalence estimates for 2019, compiled from UNAIDS and categorized as low (<1%), medium (1–4.9%) or high (≥5%) HIV prevalence [35], and country income levels in 2020 compiled from the World Bank [36]. For countries and geographic entities not available in UNAIDS 2019 data (i.e. Canada, China, India, South Korea and Taiwan), the most recent HIV prevalence estimates available were compiled from other public databases [37] and local sources [38, 39, 40], and the same prevalence cut‐offs were applied.

### 2.2 Statistical analysis

Frequencies and descriptive statistics of site‐level constraints and responses to the COVID‐19 pandemic were calculated overall and stratified by national HIV prevalence levels and country income levels. Sites that reported not providing a given service prior to the pandemic were excluded when calculating the proportion of sites whose services had been impacted by the pandemic. Fisher's exact tests were used to assess independence, with Chi‐squared tests used when exact tests could not be estimated.

All statistical analyses and descriptive mapping were performed using SAS 9.4 (SAS Institute, Cary, NC).

## 3 RESULTS

**Figure 1:**
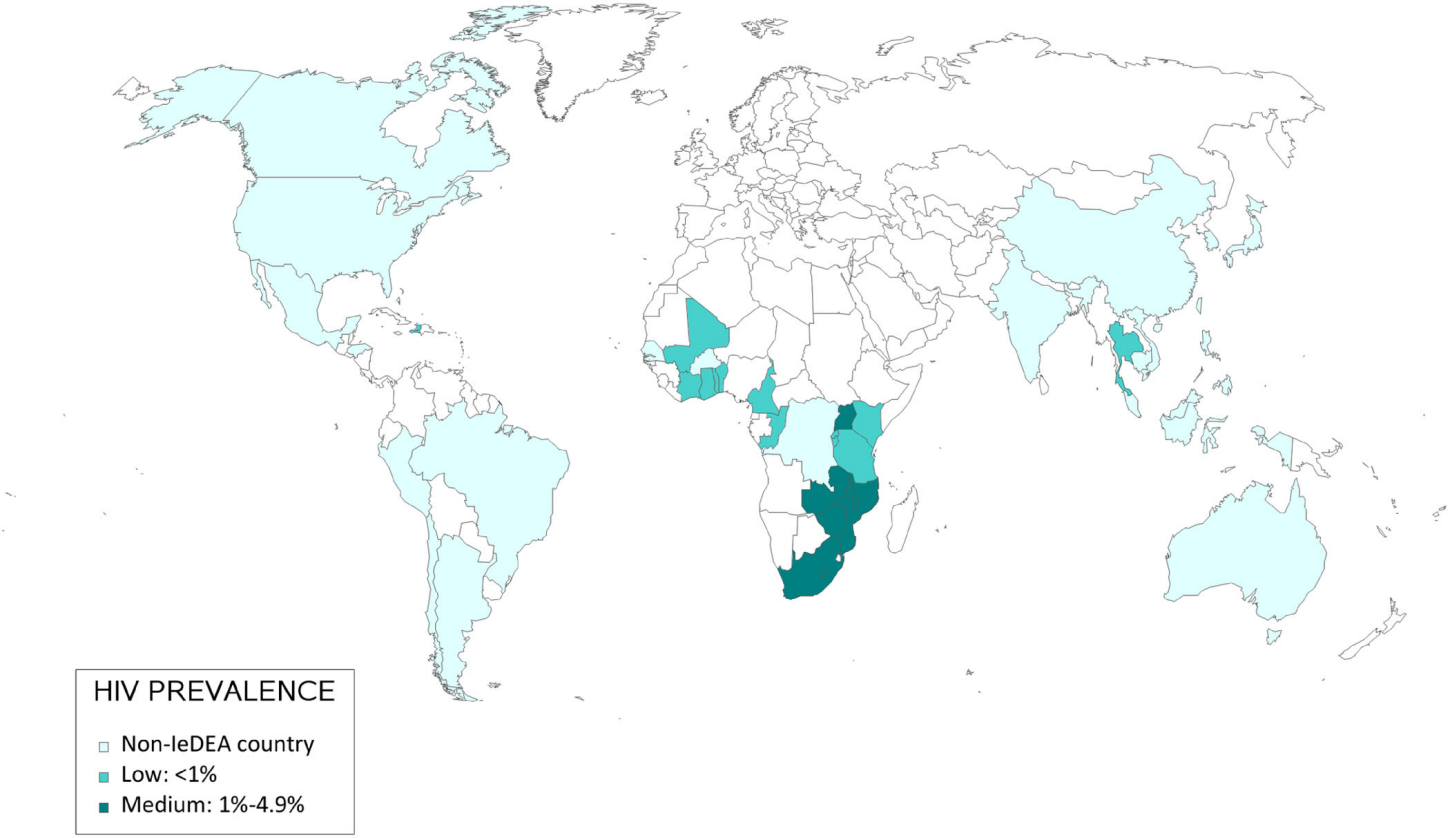
Countries represented in the International epidemiology Databases to Evaluate AIDS (IeDEA) 2020 site assessment survey, by national HIV prevalence level. ^†^Numbers in parentheses indicate the number of sites surveyed per country. ^‡^Indicates that a surveyed site represents a cohort of sites.

Out of 238 sites in 43 countries, 227 (95%) responded to the survey, and 225 (99%) in 42 countries completed questions on the pandemic's impact on HIV services and care. Sites were distributed across IeDEA's seven geographic regions (Asia‐Pacific, Central Africa, East Africa, Southern Africa, West Africa, CCASAnet and NA‐ACCORD) (Figure 1). Of 225 sites, 57 (25%) were in high HIV prevalence settings, with 86 (38%) in medium HIV prevalence settings, and 82 (36%) in low‐prevalence settings (Table 1). Most sites were in low (25%) and lower‐middle‐income countries (35%), with 17% and 22% in upper‐middle and high‐income countries, respectively.

**Table:** 

North America (*N* = 28)^†^	Caribbean, Central & South America (*N* = 9)	Central Africa (*N* = 21)	West Africa (*N* = 14)	Southern Africa (*N* = 28)	East Africa (*N* = 74)	Asia‐Pacific (*N* = 51)
Canada (2) United States (26)	Argentina (1) Brazil (3) Chile (1) Haiti (1) Honduras (1) Mexico (1) Peru (1)	Burundi (3) Cameroon (3) Democratic Republic of Congo (1) Republic of Congo (2) Rwanda (12)	Benin (2) Burkina Faso (1) Cote d'Ivoire (7) Ghana (1) Mali (1) Senegal (1) Togo (1)	Lesotho^‡^ (1) Malawi (2) Mozambique^‡^ (1) South Africa^‡^ (14) Zambia^‡^ (5) Zimbabwe^‡^ (5)	Kenya (42) Tanzania (3) Uganda (29)	Australia (18) Cambodia (2) China (1) India (3) Indonesia (4) Japan (1) Korea (1) Malaysia (6) Philippines (1) Taiwan (1) Thailand (8) Vietnam (5)

**Table 1:** Characteristics of HIV care and treatment at 225 IeDEA sites prior to the COVID‐19 pandemic, by national HIV prevalence and country income level

		National HIV prevalence				Country income level				
Site characteristic—*N* (%)	All *N* = 225	Low (<1%) *n* = 82 (36%)	Medium (1–4.9%) *n* = 86 (38%)	High (≥5%) *n* = 57 (25%)	*p*‐value^a^	Low income *n* = 57 (25%)	Lower‐middle income *n* = 79 (35%)	Upper‐middle income *N* = 39 (17%)	High income *n* = 50 (22%)	*p*‐value^a^
IeDEA region										
Asia‐Pacific	51 (23)	43 (52)	8 (9)	0 (0)	<0.0001^b^	0 (0)	11 (14)	19 (49)	21 (42)	<0.0001^b^
Central Africa	21 (9)	1 (1)	20 (23)	0 (0)		19 (33)	2 (3)	0 (0)	0 (0)	
Caribbean, Central and South America (CCASAnet)	9 (4)	8 (10)	1 (1)	0 (0)		1 (2)	1 (1)	6 (15)	1 (2)	
East Africa	74 (33)	0 (0)	45 (52)	29 (51)		29 (51)	45 (57)	0 (0)	0 (0)	
North America (NA‐ACCORD)	28 (12)	28 (34)	0 (0)	0 (0)		0 (0)	0 (0)	0 (0)	28 (56)	
Southern Africa	28 (12)	0 (0)	0 (0)	28 (49)		3 (5)	11 (14)	14 (36)	0 (0)	
West Africa	14 (6)	2 (2)	12 (14)	0 (0)		5 (9)	9 (11)	0 (0)	0 (0)	
Country income level										
Low income	57 (25)	2 (2)	23 (27)	32 (56)	<0.0001					
Lower‐middle income	79 (35)	13 (16)	55 (64)	11 (19)						
Upper‐middle income	39 (17)	17 (21)	8 (9)	14 (25)						
High income	50 (22)	50 (61)	0 (0)	0 (0)						
Facility level										
Health centre	122 (54)	37 (45)	46 (54)	39 (68)	<0.0001	36 (63)	45 (57)	7 (18)	34 (68)	<0.0001
District hospital	17 (8)	0 (0)	13 (15)	4 (7)		3 (5)	14 (18)	0 (0)	0 (0)	
Regional, provincial or university hospital	86 (38)	45 (55)	27 (31)	14 (25)		18 (32)	20 (25)	32 (82)	16 (32)	
Population served (residence)										
Predominantly urban	86 (38)	53 (65)	17 (20)	16 (28)	<0.0001	10 (18)	15 (19)	24 (62)	37 (74)	<0.0001
Predominantly rural	44 (20)	2 (2)	22 (26)	20 (35)		20 (35)	21 (27)	1 (3)	2 (4)	
Mixed urban/rural	95 (42)	27 (33)	47 (55)	21 (37)		27 (47)	43 (54)	14 (36)	11 (22)	
Patients served (age groups)										
Adults only	82 (36)	68 (83)	10 (12)	4 (7)	<0.0001	5 (9)	12 (15)	15 (39)	50 (100)	<0.0001
Adults and paediatric patients	115 (51)	1 (1)	64 (74)	50 (88)		48 (84)	58 (73)	9 (23)	0 (0)	
Paediatric patients only	28 (12)	13 (16)	12 (14)	3 (5)		4 (7)	9 (11)	15 (39)	0 (0)	
Sites reporting on pre‐pandemic ART initiation practices	172 (76)	63 (77)	64 (74)	45 (79)	0.814	51 (90)	52 (66)	27 (69)	42 (84)	0.004
Timing of ART initiation pre‐pandemic^c^										
Same day	100 (58)	20 (32)	43 (67)	37 (82)	<0.0001	34 (67)	39 (75)	13 (48)	14 (33)	<0.0001^b^
1–7 days	48 (28)	26 (41)	16 (25)	6 (13)		16 (32)	10 (19)	5 (19)	17 (41)	
8–14 days	13 (8)	7 (11)	5 (8)	1 (2)		1 (2)	2 (4)	6 (22)	4 (10)	
2–4 weeks	9 (5)	8 (13)	0 (0)	1 (2)		0 (0)	1 (2)	3 (11)	5 (12)	
>1 month	2 (1)	2 (3)	0 (0)	0 (0)		0 (0)	0 (0)	0 (0)	2 (5)	
ART counselling sessions required pre‐pandemic^c^										
0	20 (12)	14 (22)	5 (8)	1 (2)	0.0003	3 (6)	1 (1.9)	2 (7)	14 (33)	<0.0001^b^
1	95 (55)	28 (44)	35 (55)	32 (71)		32 (63)	27 (52)	16 (59)	20 (48)	
2	23 (13)	13 (21)	6 (9)	4 (9)		9 (18)	4 (8)	4 (15)	6 (14)	
3	25 (15)	3 (5)	14 (22)	8 (18)		7 (14)	13 (25)	4 (15)	1 (2)	
4 or more	9 (5)	5 (8)	4 (6)	0 (0)		0 (0)	7 (13.5)	1 (4)	1 (2)	
Pre‐pandemic ART refill frequency for stable patients^c^										
Every 1–2 months	47 (21)	28 (34)	8 (9)	11 (19)	<0.0001	4 (7)	10 (13)	19 (49)	14 (28)	<0.0001^b^
Every 3 months	141 (63)	34 (42)	67 (78)	40 (70)		48 (84)	59 (75)	15 (39)	19 (38)	
Every 4–6 months	34 (15)	19 (23)	10 (12)	5 (9)		4 (7)	9 (11)	5 (13)	16 (32)	
Other	3 (1)	1 (1)	1 (1)	1 (2)		1 (2)	1 (1)	0 (0)	1 (2)	
Pre‐pandemic viral load testing services										
No on‐site viral load testing	106 (47)	12 (15)	54 (63)	40 (70)	<0.0001	42 (74)	53 (67)	7 (18)	4 (8)	<0.0001
On‐site viral testing (within HIV unit or facility)	119 (53)	70 (85)	32 (37)	17 (30)		15 (26)	26 (33)	32 (82)	46 (92)	
Pre‐pandemic turnaround time for viral load test results										
0–7 days	100 (44)	52 (63)	33 (38)	15 (26)	<0.0001	9 (16)	29 (37)	22 (56)	40 (80)	<0.0001^b^
14 days	50 (22)	15 (18)	29 (34)	6 (11)		9 (16)	26 (33)	8 (2)	7 (14)	
15–30 days	58 (26)	13 (16)	19 (22)	26 (46)		29 (51)	18 (23)	8 (2)	3 (6)	
30–60 days	14 (6)	1 (1)	4 (5)	9 (16)		9 (16)	5 (6)	0 (0)	0 (0)	
Not available	3 (1)	1 (1)	1 (1)	1 (2)		1 (2)	1 (1)	1 (3)	0 (0)	

^a^ Fisher's exact test.

^b^ Chi‐squared test.

^c^ Denominators exclude sites that did not report ART initiation services prior to the pandemic.

### 3.1 Site characteristics

Of 225 sites completing the survey, 54% were health centres, 8% were district hospitals and 38% were tertiary regional/provincial or university teaching hospitals. One‐fifth reported serving a predominantly rural population, with 38% serving a predominantly urban population and 42% serving a mixed urban/rural population. Half of the sites reported serving both adult and paediatric patients, including almost all sites in high HIV prevalence settings (88%) and low‐income countries (84%). In contrast, most sites in low‐prevalence settings (83%) and high‐income countries (100%) served adult patients only.

### 3.2 Routine pre‐pandemic ART initiation practices and VL testing capacity at participating sites

All 225 sites reported initiating patients on treatment before the pandemic and 172 provided information on routine pre‐pandemic ART initiation practices, with 58% reporting that they had typically initiated patients on ART on the same day as enrolment in HIV care, including 32% of sites in low‐prevalence settings, 67% in medium‐prevalence settings and 82% in high‐prevalence settings. Most sites in high‐prevalence settings (71%) and low‐income countries (63%) reported that patients were typically required to complete a single pre‐ART counselling session prior to the pandemic, whereas sites in low‐prevalence settings and high‐income countries were more likely to report that no counselling sessions or two or more such sessions had been required. The majority of clinics surveyed (63%) reported that patients stable on ART had received 3‐month ART supplies before the pandemic, with 3‐month refill frequencies more commonly reported in high‐prevalence and low‐income settings (70% and 84%, respectively) compared with low‐prevalence (42%) and high‐income (38%) settings.

Just over half of sites surveyed (53%) reported being able to provide on‐site VL testing prior to the pandemic, ranging from 85% of sites in low‐prevalence settings to 30% of sites in high‐prevalence settings, with VL testing capacity strongly associated with country income levels (26% of sites in low‐income countries provided on‐site testing, compared with 92% of sites in high‐income countries). Comparable differences across settings were observed in VL testing turnaround times, with 63% of sites in low‐prevalence settings (80% in high‐income countries) reporting that results were received within 1 week, compared with 26% and 16% of sites in high‐prevalence settings and low‐income countries, respectively.

### 3.3 Changes in clinic environment or operations related to the COVID‐19 pandemic

Changes in clinic operations reported by survey respondents are shown in Table 2. Overall, 75% of sites reported that their clinic location had been subject to some form of pandemic‐related restrictions on travel, service provision or other business operations. Sites in high HIV prevalence settings reported longer periods of lockdowns and restrictions, with 58% reporting lockdowns lasting 4–7 months (56% of sites in low‐income countries). Only 19% of sites in low‐prevalence settings and 12% in high‐income countries reported lockdowns of this duration. Few sites (8%) reported completely suspending HIV service provision in response to the pandemic, with service suspensions being more common in settings with low HIV prevalence (17%), compared with medium‐ and high‐prevalence settings (4% and 0%, respectively).

**Table 2:** Changes in clinic operations during the first year of the COVID‐19 pandemic at 225 IeDEA sites, by national HIV prevalence and country income level

		National HIV prevalence				Country income level				
Change in clinic environment or operations *N* (%)	All *N* = 225	Low (<1%) *n* = 82	Medium (1–4.9%) *n* = 86	High (≥5%) *n* = 57	*p*‐value^a^	Low income *n* = 57	Lower‐middle income *n* = 79	Upper‐middle income *N* = 39	High income *n* = 50	*p*‐value^a^
Geographic area surrounding this HIV clinic subject to any form of COVID‐19 restrictions on travel, service provision or business operations	168 (75)	64 (78)	52 (60)	52 (91)	<0.0001	45 (79)	52 (66)	30 (77)	41 (82)	0.167
Duration of lockdowns/restrictions at sites subject to COVID‐19 restrictions										
< = 1 month	11 (7)	5 (8)	5 (10)	1 (2)	0.001	3 (7)	3 (6)	1 (3)	4 (10)	0.001^b^
2–3 months	54 (32)	27 (42)	15 (29)	12 (23)		10 (22)	11 (21)	13 (43)	20 (49)	
4–7 months	59 (35)	12 (19)	17 (33)	30 (58)		25 (56)	21 (40)	8 (27)	5 (12)	
Ongoing	23 (14)	13 (20)	7 (14)	3 (6)		1 (2)	8 (15)	6 (20)	8 (20)	
Do not know/recall^c^	21 (12)	7 (10)	8 (15)	6 (11)		6 (13)	9 (17)	2 (7)	4 (10)	
HIV services suspended	17 (8)	14 (17)	3 (4)	0 (0)	0.0001	0 (0)	7 (9)	6 (15)	4 (8)	0.016
Duration of service suspension among sites suspending HIV services										
< = 1 month	3 (18)	3 (21)	0 (0)	0 (0)	0.232	0	1 (14)	1 (17)	1 (25)	0.802
2–3 months	6 (35)	6 (43)	0 (0)	0 (0)		0	2 (29)	2 (33)	2 (50)	
4–7 months	6 (35)	4 (29)	2 (67)	0 (0)		0	3 (43)	3 (50)	0 (0)	
Ongoing at the time of survey completion	2 (12)	1 (7)	1 (33)	0 (0)		0	1 (14)	0 (0)	1 (25)	
Negative impacts on clinic operations										
Decreased hours or days of service delivery for HIV patients	58 (26)	25 (31)	23 (27)	10 (18)	0.217	11 (19)	23 (29)	15 (39)	9 (18)	0.094
HIV care providers reassigned to assist with the COVID‐19 response	83 (37)	38 (46)	17 (20)	28 (49)	0.0001	18 (32)	20 (25)	22 (56)	23 (46)	0.004
Reduced availability of HIV care providers due to COVID‐19‐related illness, self‐isolation or quarantine	88 (39)	38 (46)	24 (28)	26 (46)	0.024	24 (42)	25 (32)	21 (54)	18 (36)	0.124
Reconfiguration of hospital/clinic space to accommodate COVID‐19‐related services	118 (52)	54 (66)	40 (47)	24 (42)	0.008	29 (51)	35 (44)	24 (62)	30 (60)	0.208
Interruptions or changes in data recording (paper or electronic records) related to clinical management of patients	47 (21)	21 (26)	9 (11)	17 (30)	0.007	13 (23)	11 (14)	14 (36)	9 (18)	0.054
Withdrawal/suspension of activities of non‐governmental partners that support care provision in the clinic (*n* = 177)^d^	56 (32)	26 (42)	14 (19)	16 (37)	0.013	15 (36)	11 (17)	17 (50)	13 (37)	0.004
Any of the above negative impacts	172 (76)	71 (87)	54 (63)	47 (83)	0.001	46 (81)	49 (62)	36 (92)	41 (82)	0.001
Adaptive clinic responses										
Increased use of personal protective equipment (masks, gloves, gowns, etc.) by HIV clinic staff	208 (92)	77 (94)	77 (90)	54 (95)	0.503	53 (93)	72 (91)	35 (90)	48 (96)	0.693
Increased use of telemedicine (i.e. consultations by phone/web) in HIV‐related care	126 (56)	70 (85)	33 (38)	23 (40)	<0.0001	20 (35)	31 (39)	25 (64)	50 (100)	<0.0001

^a^ Fisher's exact test.

^b^ Chi‐squared test.

^c^ Do not know/recall responses excluded from significance testing.

^d^ Denominators (in parentheses) exclude sites that did not offer a given service prior to the pandemic.

Most sites (76%) reported at least one negative impact of the pandemic on clinic operations, with no differences across HIV prevalence or income levels. Negative impacts included: the reconfiguration of hospital/clinic space to accommodate COVID‐19‐related services (52%), reduced provider availability because of illness, self‐isolation or quarantine (39%), reduced provider availability because of reassignment to assist with the COVID‐19 response (37%), withdrawal or suspension of support from non‐governmental partners engaged in HIV care (32%), reduced hours/days for the provision of HIV services (26%) and interruptions in medical record‐keeping or data entry (21%).

Almost all sites (92%) reported increased use of personal protective equipment in response to the pandemic, with no significant differences across settings. In contrast, sites in low‐prevalence settings (85%) and high‐income countries (100%) were more than twice as likely as sites in medium‐ and high‐prevalence settings and low‐income countries to report increased use of telemedicine (i.e. consultations by telephone or web‐based conferencing) in the provision of HIV‐related care.

### 3.4 Effects of the pandemic on HIV service provision

Pandemic‐related changes in HIV service provision are shown in Table 3. Overall, 26% of sites reported suspending HIV testing and diagnostic services, and 10% reported suspending the enrolment of new patients into HIV care. Almost half (42%) of sites reported suspending or postponing non‐urgent appointments for HIV patients, primarily in settings with low HIV prevalence settings (65%) and upper‐middle‐ and high‐income countries (64%).

**Table 3:** Changes in HIV services and programmes during the first year of the COVID‐19 pandemic at 225 IeDEA sites, by national HIV prevalence and country income level

		HIV prevalence				Country income level				
Service/programme attribute^a^—*N* (%)	All *N* = 225	Low (<1%) *n* = 82	Medium (1–4.9%) *n* = 86	High (≥5%) *n* = 57	*p*‐value^b^	Low income *n* = 57	Lower‐middle income *n* = 79	Upper‐middle income *N* = 39	High income *n* = 50	*p*‐value^b^
Disruptions in services operated by the HIV clinic										
HIV testing or diagnostic services suspended/reduced (*n* = 215)^a^	56 (26)	25 (32)	13 (16)	18 (34)	0.020	22 (41)	13 (18)	9 (24)	12 (24)	0.037
New patient enrolments suspended	23 (10)	10 (12)	7 (8)	6 (11)	0.725	6 (11)	9 (11)	7 (18)	1 (2)	0.073
Non‐urgent appointments for HIV patients suspended or postponed	94 (42)	53 (65)	23 (27)	18 (32)	<0.0001	18 (32)	19 (24)	25 (64)	32 (64)	<0.0001
ART clinics suspended	17 (8)	12 (15)	4 (5)	1 (2)	0.012	1 (2)	5 (6)	6 (15)	5 (10)	0.067
ART initiation services suspended	13 (6)	8 (10)	3 (4)	2 (4)	0.194	2 (4)	5 (6)	5 (13)	1 (2)	0.190
Adaptive measures introduced by the HIV clinic										
Same‐day/rapid ART initiation expanded (*n* = 200)^a^	67 (30)	20 (24)	27 (31)	20 (35)	0.661	22 (41)	28 (36)	7 (26)	10 (24)	0.265
Adherence counselling streamlined (*n* = 214)^a^	61 (27)	22 (27)	23 (27)	16 (28)	0.926	18 (32)	22 (29)	13 (35)	8 (19)	0.364
Community ART pick‐up points designated	52 (23)	10 (12)	17 (20)	25 (44)	<0.0001	18 (32)	19 (24)	10 (26)	5 (10)	0.049
Patients given extra ART supplies to reduce refill frequency	182 (81)	55 (67)	73 (85)	54 (95)	0.0001	50 (88)	63 (80)	34 (87)	35 (70)	0.097
Viral load testing services										
HIV VL sample collection suspended (*n* = 223)^a^	49 (22)	23 (28)	15 (17)	11 (19)	0.226	15 (27)	18 (23)	7 (18)	9 (18)	0.710
HIV VL samples not accepted by laboratory (*n* = 221)^a^	26 (12)	9 (11)	11 (13)	6 (11)	0.897	11 (20)	8 (10)	4 (10)	3 (6)	0.159
Longer turn‐around time for VL results (*n* = 222)^a^	92 (41)	38 (46)	30 (35)	24 (42)	0.331	29 (52)	32 (41)	14 (36)	17 (35)	0.279
Other disruptions (staffing shortages and lack of transport)	5 (2)	2 (2)	3 (4)	0 (0)	0.448	0 (0)	2 (3)	1 (3)	2 (4)	0.492
Stockouts										
PrEP (*n* = 179)^a^	11 (6)	2 (3)	7 (10)	2 (5)	0.250	4 (9)	5 (7)	2 (9)	0 (0)	0.129
HIV test kits (*n* = 207)^a^	17 (8)	4 (6)	9 (11)	4 (7)	0.562	6 (11)	9 (12)	2 (6)	0 (0)	0.092
First‐line ART (*n* = 221)^a^	12 (5)	4 (5)	5 (6)	3 (5)	1.000	3 (5)	6 (8)	2 (5)	1 (2)	0.625
Second‐line ART (*n* = 220)^a^	25 (11)	5 (6)	6 (7)	14 (25)	0.002	12 (22)	9 (12)	3 (8)	1 (2)	0.012
Third‐line ART (*n* = 139)^a^	14 (10)	3 (4)	7 (16)	4 (17)	0.049	6 (22)	5 (17)	2 (6)	1 (2)	0.011
Supplies for viral load testing (*n* = 209)^a^	45 (22)	12 (16)	18 (22)	15 (29)	0.221	23 (44)	15 (20)	4 (11)	3 (7)	<0.0001
Disruptions in community‐based services, including services operated by partners										
Withdrawal/suspension of non‐governmental partner support for community‐based programmes for enrolled patients (*n* = 162)^a^	65 (40)	31 (59)	14 (22)	20 (46)	0.0001	13 (31)	19 (31)	19 (66)	14 (48)	0.007
Community‐based HIV testing suspended (*n* = 165)^a^	117 (71)	38 (72)	40 (66)	39 (77)	0.462	31 (59)	40 (58)	20 (69)	7 (33)	0.735
Community‐based ART refills suspended (*n* = 142)^a^	59 (42)	11 (28)	29 (50)	19 (43)	0.077	16 (36)	30 (59)	12 (55)	1 (4)	<0.0001
Community‐based support group meetings/activities suspended (*n* = 169)^a^	136 (81)	47 (84)	50 (78)	39 (80)	0.734	35 (76)	52 (81)	23 (79)	26 (87)	0.722
Community‐based tracing of patients lost to follow‐up suspended (*n* = 172)^a^	98 (57)	25 (58)	36 (49)	37 (67)	0.105	31 (59)	40 (58)	20 (69)	7 (33)	0.091

^a^ Denominators (in parentheses) exclude sites that did not offer a given service prior to the pandemic.

^b^ Fisher's exact test.

While few sites (8%) reported suspending ART services, such suspensions were more commonly reported by sites in low‐prevalence settings (15%), compared with medium‐ (5%) or high‐prevalence (2%) settings. Among sites that offered ART initiation services, few (6%) reported suspending these services. In contrast, many reported the introduction or expansion of adaptive measures to mitigate the pandemic's impacts on treatment adherence, with 81% of sites reporting ever giving patients additional supplies of ART to reduce the frequency of refills and 23% reporting ever designating community ART pick‐up points to reduce patients’ travel burden. Both these mitigation strategies were more commonly reported by sites in high‐prevalence settings (95% and 44%, respectively), compared with medium‐ (85% and 20%, respectively) and low‐prevalence settings (67% and 12%, respectively). Other adaptive measures reported by clinics included the expansion of same‐day ART initiation (30%) and streamlined ART adherence counselling (27%), with no significant differences across settings.

Across all settings, few sites reported stockouts of pre‐exposure prophylaxis (PrEP) medications for HIV prevention (6%), HIV test kits (8%) or first‐line antiretroviral medications (5%). In contrast, among sites providing second‐ and third‐line ART, about 10% of sites reported stockouts of these medications, which were approximately four times as likely in high‐prevalence settings, compared with low‐prevalence settings. Stockouts were significantly more prevalent in low‐ and lower‐middle‐income countries than in upper‐middle‐ and high‐income countries.

Survey respondents reported negative impacts of the pandemic on HIV VL testing services, including longer turnaround times for results (41%), suspension of blood draws for VL testing (22%) and HIV VL samples not being accepted by laboratories (12%). In addition, 22% of sites conducting VL testing prior to the pandemic reported supply stockouts. VL testing disruptions did not differ by HIV prevalence or country income levels.

Among sites reporting the existence of various community‐based services for PWH prior to the pandemic, including services operated by other partners, 71% reported that community‐based HIV testing services had been suspended at some point during the pandemic, and 81% reported the suspension of community‐based support group meetings/activities. Other pandemic impacts on community‐based services for PWH included the suspension of tracing programmes (57%), withdrawal of non‐governmental partner support (40%) and suspension of community‐based ART refill programmes (42%). Few pandemic‐related impacts on community‐based programmes for PWH differed across HIV prevalence settings or country income levels.

## 4 DISCUSSION

With data from 225 HIV treatment sites across 42 countries at the end of the first year of the COVID‐19 pandemic, this study found that most had experienced disruptions in clinic operations and in the provision of HIV care. Such disruptions were reported by sites across high‐, medium‐ and low‐HIV prevalence settings and country income levels, reinforcing concerns raised by modelling studies [9, 11, 12, 13] and observational research [41, 42, 43, 44, 45, 46, 47, 48, 49] about the potential of COVID‐19 to reverse progress towards 95‐95‐95 UNAIDS targets to end the HIV epidemic, particularly in settings with a high HIV burden [50].

Our study included several noteworthy findings. While clinics in high HIV prevalence settings were most likely to report being subject to pandemic‐related restrictions affecting travel, service provision or business operations, they were less likely than clinics in low‐prevalence settings to report suspending or postponing non‐urgent appointments for HIV patients or having to reconfigure clinic space to accommodate COVID‐19‐related services. Clinics in high‐prevalence settings were also less likely to report ever suspending ART clinics, and, in contrast with low‐ and medium‐prevalence settings, none of the clinics in high‐prevalence settings reported ever suspending HIV care and treatment services. Additionally, clinics in high‐prevalence settings were more likely to report the adoption of mitigation strategies (e.g. establishing ART pick‐up points in the community, providing additional stocks of ART, expanding same‐day ART initiation and reducing adherence counselling requirements) to support patient adherence and reduce barriers to care. The resilience of clinics in high‐prevalence settings may reflect the adoption of DSD strategies, such as community distribution and multi‐month dispensing of ART, prior to the pandemic, as well as investments in strengthening the efficiency of ART service delivery in these settings—a capacity that could be leveraged and expanded to support uninterrupted treatment during the first year of COVID‐19 [51].

While these findings are encouraging, the results of our study also point to resource disparities across countries that have implications for the continuity and quality of HIV care and its effectiveness in ensuring sustained viral suppression among PWH who are engaged in care. Although few clinics in low‐income settings in our survey reported suspending routine ART clinics or ART initiation for new patients, well over one‐third suspended HIV testing services, possibly reflecting resource constraints for laboratory and diagnostic services in these settings. We also observed significant disparities in the adoption of telemedicine for the provision of HIV care, with sites in high‐ and medium‐HIV prevalence settings being less than half as likely as sites in low‐prevalence settings to report increasing their use of telephone and web‐based consultations for HIV patients. Correlated with country income levels, these disparities may reflect a range of barriers and challenges for telemedicine adoption, from socio‐economic, digital literacy and linguistic barriers among patients to infrastructure, technology and regulatory obstacles for health systems [52]. While clinics in high‐prevalence settings were less likely to report suspending HIV services and appointments, other care‐seeking barriers (e.g. lockdown restrictions, transportation and financial barriers, and concerns about COVID‐19 exposure) may have presented insurmountable obstacles for patients in these settings, and early data from diverse settings have highlighted sharp decreases in healthcare‐seeking for HIV‐related services early in the pandemic, including HIV prevention [49, 53], diagnosis [24, 41, 42, 44, 45, 46, 47, 48, 53, 54, 55] and treatment [24, 43, 44, 53, 55, 56], as well as diagnostics and treatment for other infectious diseases and chronic conditions [55, 56, 57, 58]. While some studies in sub‐Saharan Africa have reported rebounds in HIV testing and ART initiation [24, 59], our findings related to stockouts of second‐ and third‐line ART regimens in high‐prevalence and low/lower‐income settings are concerning, as the lack of such essential medicines may result in setbacks for both HIV treatment and prevention.

Given the importance of VL monitoring for detecting therapeutic failure and ensuring timely adherence support and regimen switching for the health of the individual with HIV and the reduced likelihood of transmission [60], disruptions in HIV VL testing services are of concern. Ranging from stockouts of essential supplies to suspension of VL testing services, laboratories not accepting HIV VL samples and longer turnaround times for results, such disruptions may reflect supply chain problems [2, 3, 61, 62, 63], as well as the reallocation of resources from HIV programmes to the COVID‐19 response [57, 58, 63, 64]. While these disruptions were reported across all settings, they are of particular concern in settings with high HIV prevalence and in low/lower‐income countries, given that the turnaround time for VL test results was already markedly longer in these settings before the pandemic. These gaps underscore the need for investment in integrated laboratory systems to increase access to critical diagnostics without having one infectious disease displace others [58, 63].

Our study had several limitations. Firstly, as the COVID‐19 pandemic catalyzed the adoption of new service delivery strategies that were not explored in prior IeDEA surveys, it is difficult to quantify the magnitude of some changes reported by respondents. Secondly, sites participating in IeDEA may not be representative of HIV service delivery within a country or region, and in some settings, IeDEA sites may be better resourced than many other HIV care and treatment sites, meaning that the pandemic's impact on HIV care could be underestimated in our survey. Accordingly, our findings may not be generalizable to all HIV clinical care settings in countries with low‐, medium‐ and high‐HIV prevalence or within country income groups. In addition, because disruptions to supply chains may have persisted or worsened after the end of the first year of the pandemic when our survey was conducted, the consequences for ART services and laboratory monitoring may not be fully captured. Finally, IeDEA's global site assessment surveys rely on self‐report by survey respondents, which may have introduced both selection and recall biases. While the high response and survey completion rate may help mitigate these biases, it should be noted that the survey was implemented during a period when healthcare systems and providers were at different phases of the pandemic response and when many changes in practice were being introduced to mitigate pandemic‐related risks and burdens.

Providing early data on how COVID‐19 has affected the availability of HIV services across a geographically diverse group of HIV care and treatment sites, our study complements other recent studies exploring the pandemic's impact on HIV testing, ART initiation, routine visits for HIV treatment, VL monitoring and viral suppression [24, 25, 59]—studies that found minimal changes in routine HIV care‐seeking during 2020, along with rebounds following initial decreases in HIV testing and ART initiation. While the expansion of adaptive measures reported in our survey may explain these encouraging findings, our study also underscores the need for ongoing monitoring of service disruptions, as well as research to identify capacity and services—from integrated laboratory systems and telemedicine infrastructure to supply chains and community support groups—that need rebuilding and strengthening in the wake of the pandemic. Further research within and across countries is needed to assess the impact of the pandemic on clinical and programmatic outcomes among people living with and at risk for HIV and to examine the role of site‐level adaptive measures, such as the use of telemedicine, multi‐month dispensing of ART medications or the establishment of community‐based ART pick‐up points, in averting treatment interruptions and ensuring the provision of person‐centred HIV care .

## 5 CONCLUSIONS

While the first year of the COVID‐19 pandemic resulted in widespread HIV service delivery disruptions, many IeDEA sites in high HIV prevalence and low‐resource settings introduced or expanded measures to minimize treatment interruption and care disengagement. Disruptions in VL testing and ART supplies in these settings raise concerns about the ongoing consequences of the pandemic on the availability, quality and comprehensiveness of HIV care.

## COMPETING INTERESTS

KN Althoff is a consultant to the All of Us Research Program and TrioHealth, Inc. D Nash reports a research grant from Pfizer to assess the effect of COVID‐19 vaccination on long‐haul COVID. All authors have no competing interests.

## AUTHORS’ CONTRIBUTIONS

EB, RA, DN, BM, FM, SND and CWW conceptualized the study and designed survey questions. FM coordinated data collection. EB performed the data analysis and drafted the manuscript. All authors participated in the interpretation of the results, revision of the manuscript, and have read and approved the final manuscript.

## Supporting information




**Supporting Information**: Acknowledgments and members of the International epidemiology Databases to Evaluate AIDS.

## Data Availability

The data that support the findings of this study are available from the corresponding author upon reasonable request. Individuals who wish to request access to data from the IeDEA consortium for research purposes may submit a concept proposal, which is detailed at https://www.iedea.org/.
