# Supplementary material for: Service delivery challenges in HIV care during the first year of the COVID‐19 pandemic: results from a site assessment survey across the global IeDEA consortium
Source: J Int AIDS Soc. 2022 Dec 11;25(12):e26036. doi: 10.1002/jia2.26036 (PMC9742047; doi:10.1002/jia2.26036)
Supplement: Supplementary file 1 — Supporting Information: Acknowledgments and members of the International epidemiology Databases to Evaluate AIDS. [file JIA2-25-0-s001.docx]

**Acknowledgements**

**Funding:** The International Epidemiology Databases to Evaluate AIDS (IeDEA) is supported by the U.S. National Institutes of Health’s National Institute of Allergy and Infectious Diseases, the Eunice Kennedy Shriver National Institute of Child Health and Human Development, the National Cancer Institute, the National Institute of Mental Health, and the National Institute on Drug Abuse: Asia-Pacific, U01AI069907; CCASAnet, U01AI069923; Central Africa, U01AI096299; East Africa, U01AI069911; NA-ACCORD, U01AI069918; Southern Africa, U01AI069924; West Africa, U01AI069919. This work is solely the responsibility of the authors and does not necessarily represent the official views of any of the institutions mentioned above.

The authors would like to thank the following site investigators, clinicians and data managers who distributed and completed the 2020 IeDEA Site Assessment, along with members of the IeDEA Site Assessment Working Group.

**IeDEA Asia Pacific**

Chidchon Chansilpa, Trevor Dougherty, Azar Karminia, Matthew Law, Jeremy Ross, Annette Sohn.

**Australia:** Ivette Aguirre, David Baker, Mark Bloch, Safaa Cabot, Andrew Carr, Deborah Couldwell, Sian Edwards, Beng Eu, Heather Farlow, Robert Finlayson, Manoji Gunathilake, Cherie Hazlewood, Jennifer Hoy, Julian Langton-Lockton, Jacqueline Le, Elizabeth Leprince, Ariane Minc, Richard Moore, Maree O'Sullivan, Norm Roth, Dianne Rowling, Darren Russell, Nathan Ryder, Craig Saunders, Julie Silvers, David J. Smith, David Sowden, Grant Sweeney, Lynn Tan, Ricard Teague, David Templeton, Caroline Thng, Ian Woolley. **Cambodia:** Vohith Khol, Penh Sun Ly. **China:** Tsz Hei Li, Lee Man Po. **India:** Aarti Kinikar, Nagalingeswaran Kumarasamy, Sanjay Mundhe, Sanjay Pujari, Shashikala Sangle, Smita Nimkar. **Indonesia:** Madelein Jassin, Nia Kurniati, Tuti Parwati Merati, Dina Muktiarti, Rizqi Amalia, Ni Made Dewi Dian Sukmawati, Ketut Dewi Kumara Wati, Evy Yunihastuti. **Japan:** Junko Tanuma. **Republic of Korea:** Jun Yong Choi. **Malaysia:** Raja Iskandar Shah Raja Azwa, Chan Kwai Cheng, Yasmin Mohamed Gani, Thahira Jamal Mohamed, Fong Siew Moy, Revathy Nallusamy, Mohamad Zulfahami Mohd Nor, Nuraini Rudi, Wong Peng Shyan, Nik Khairulddin Nik Yusoff. **The Philippines:** Rossana Ditangco. **Taiwan:** Yu-Jiun Chan, Pei-Chieh Wu, Ping-Feng Wu. **Thailand:** Anchalee Avihingsanon, Romanee Chaiwarith, Kulkanya Chokephaibulkit, Suwimon Khusuwan, Sasisopin Kiertiburanakul, Pope Kosalaraksa, Pagakrong Lumbiganon, Pradtana Ounchanam, Thanyawee Puthanakit, Supattra Rungmaitree, Nuttarika Solai, Tavitiya Sudjaritruk. **Vietnam:** Vu Thien An, Do Duy Cuong, Chau Viet Do, Bui Vu Huy, Tuan Quy, Kinh Van Nguyen, Luan Nguyen, Van Lam Nguyen, Yen Thi Nguyen, Vuong Minh Nong, Huu Khanh Truong, Ngo Thi Thu Tuyen.

**IeDEA Caribbean, Central and South America (CCASAnet)**

Catherine C. McGowan, Stephany Duda, Fernanda Maruri, C. William Wester

**Argentina:** Florencia Cahn, Pedro Cahn, Carina Cesar, Valeria Fink, Omar Sued. **Brazil:** Lara Coelho, Daisy Maria Machado, Jorge Pinto. **Chile:** Marcelo Wolff. **Haiti:** Vanessa Rouzier. **Honduras:** Denis Padgett. **Mexico:** Brenda Crabtree Ramírez. **Peru:** Eduardo Gotuzzo.

**Central Africa IeDEA**

Ellen Brazier, Denis Nash.

**Burundi:** Jérémie Biziragusenyuka, Patrick Gateretse, Pelagie Nimbona, Olive Niyonkuru, Christelle Twizere.

**Cameroon:** Rogers Ajeh, Surreng Anicetus, Amadou Djenabou, Priscilla Enow, Eyongetah Mbu, Martin Manga, Mercy Ndobe, Judith Nasah, Elle Nathalie Syntyche Ekossono, Mireille Teno Bouseko. **Democratic Republic of the Congo:** Faustin Kitetele, Patricia Lelo. **Republic of Congo**: Merlin Isidore Justin Diafouka, Adolphe Mafoua, Dominique Mahambou Nsonde. **Rwanda:** Uitonze Aime Maurice Bihira, Marie Chantal Dusabe, Rosine Feza, Jean Claude Habanabashaka, Viateur Habumuremyi, Ernestine Igizeneza, Anne Marie Kamigisha, Gallican Kubwimana, Gilbert Maniriho, Gilbert Mbaraga, Benjamin Muhoza, Jeanne Mukakarangwa, Joyce Mukamana, Patricie Mukanyirigira, Yvone Claude Mukeshimana, Athanase Munyaneza, Gad Murenzi , Jacqueline Musaninyange, Jules Ndumuhire Nyiraneza, Fidele Ntarambirwa, Marie Louise Nyiraneza, Josette Tuyishime, Yvonne Tuyishimire, Alexis Ubandutira, Florance Umugiraneza, Rosine Umugwaneza, Olive Uwamahoro, Pauline Uwamahoro, Marie Victoire Uwambaje, Clarisse Uwimpuhwe, Siphora Uwiragiye.

**East Africa IeDEA**

Yee Yee Kuhn, Beverly Musick, Kara Wools-Kaloustian.

**Kenya:** Felix Adera, BeatricecAdhiambo, Khaemba Aggrey, Daniel Akadikor, Felix Ambulla, Dorah Apiyo, Patrick Ariya, Naftal Atemba, Fridah Ayodi., Chirchir Benard, Maureen Bett, Serafine Birgen, Rael Bwalei, Nancy Chebon, Valentine Jirry Chebor, Philip Chebuiywo, Jacline Chemutai, Emily Chepkorir, Carolyne Chepseba, John Chirchir, John Chirchir, Lameck Diero, Benard Dukwa, Alice Elphas, Tom Etyang, Agnes Idiama, Ann Jebichuko, Delvine Jepchumba, Churchill Juma, Maureen Juma, Sheila Juma., Julie Kadima, Rose Karani, Christopher Keitany, Pricilla Keter, Lucy Kiavoga, Harrison Kibet, Ruth Kimutai, Mutai Kiplagat, Wilfred Kiprono, Nicholas Kogei Kipruto, Asenath Kirimi, Zeddy Koech, Carolyne Kosgei, Karen Kutto, Mildred Kweyu, Ephraim Kenneth Liech, Milka Limo, Rose Maina, Priscah Marumbu, Agnes Masese, Patricia Mochotto, Omudeck Molly, Tom Momanyi, John W. Murutu, Praxidis Mwanda, Lillian Ndakalu, Rose N. Nderitu, Sarah Obatsa, Fredrick Obiga, Moses Oboya, Joseph Odhiambo, George Olaya, Oscar Omanyala, Christine Oray, Molly Otieno, Modesta Toto Otwane, Paul Ouma, Charles Owuor, Doris Tutu Pepela, Collins Pessah, Evans Rotich, Edwin K. Rotich, Titus C. Rutto, Monica Shikuku, Rose Naliaka Sibweche, Robert Wanyonyi Simiyu, Hellen Siria, Michael Some, Winnie Cherotich Songok, Immaculate Tanui, Grace Wafula, Rebecca Wambura, Ellah Wanjala, Carolyne Wanyama, Hellen Wanyonyi, Emmanuel Woyakapel, Wandera Zelbabel, Judy, Kiprop, Beatrice, Leah, Dominic, Tallam. **United Republic of Tanzania:** Dikengela Gwimo, Ester Kinyota, Jerome Lwali, Rita Lyamuya, Richard Machemba, Julia Mathias, Lilian Mkombachepa, Athuman Mokiwa, Ombeni Mushi, Charles Ndunguru, Kapella Ngonyani, Charles Nyaga, Happiness Ruta, Mark Urassa. **Uganda:** James Akanyihayo, Arnold Arinaitwe, Jesca Batuuka, Walusimbi Birungi, John Nyanzi Bugembe, Ahmed Ddungu, Kato Francis, Bangira Imran, George William Kafuuma, John Bosco Kalulue, Grace Kanaabi, Michale Kanyesigye, Godfery Karuhanga, Charles Kasozi, Godfrey Kasule, Assumpta Katusime, Donozio Kibalama, Donozio Kibalama, Simon Peter Kimera, Namatovu Kulusumu, Yusuf Lule, Isaac Lwanga, Margaret Mluindwa, Jemba Moses, Sseremba Mubarak, Daniel Muggaga, Evelyn Mukalazi, Joseph Muleebwa, Derick Mulema, Ivan Musisi, John Muwawu, Winnie Muyindike, Dick Mwaka, Milly Naava, Immaculate Nabiyki, Agnes Nabusulwa, Dorah Nakabugo, Esther Nakamya, Daisy Nakanwagi, Oliver Nakato, Lydian Nakayi, Patience Nakigozi, Juliet Nakku, Juliet Nakuya, Justine Nakyomu, Joan Namayanja, Sarah Namirembe, Juliet Namugumya, Ezereth Namukasa, Viola Namulindwa, Irene Nankya, Grace Mugagga Nannyondo, Harriet Nansamba, Denis Nansera, Brenda Nanyanzi, Esther Celina Nanyonjo, Irene Nayiga, Isaac Opira, Noela C. Owarwo, Sserunkuma Resty, Haruna Semuwemba, Julius Senoga, Gerald Sseguya, John Paul Ssekyewa, Matthew Ssemakadde, Jonah Tebajjwa, Doreen Tugumisirize, Robinah Tushemerirwe, Kawuki Waliyi, Fenehance, Medard.

**IeDEA North American AIDS Cohort Collaboration on Research and Design (NA-ACCORD)**

Richard Moore, Keri Althoff, Aimee Freeman.

**Canada:** Jennifer Bishop, M J Gill, Mona Loutfy, Graham Smith. **United States of America:** Laura Bamford, Anthony Black, Asia Brice, Sheldon Brown, Jonathan Colasanti, Piper Duarte, Cynthia Firnhaber, Matthew Goetz, Chris Grasso, Barbara Gripshover, Michael Horberg, Rita Kelly, Ken Levine, Mitchell Luu, Vincent Marconi, Karen Maroney, Kenneth Mayer, Angel Mayor, Catherine McGowan, Richard Moore, Ami Multani, Sonia Napravnik, Ank Nijhawan, Richard Novak, Frank Palella, Maria C. Rodriguez, Mia Scott, Ellen Tedaldi, James Willig.

**IeDEA Southern Africa**

Morna Cornell, Mary-Ann Davies, Matthias Egger, Andreas Haas

**Lesotho:** Monkoe Bereng, Maleshoane Kalake, Keketso Lenela, Relebohile Seretse. **Malawi:** Matthews Chintenga, Jane Chiwoko, Joe Gumulira, Jacqueline Huwa, Rafique Maluwa, Beatrice Matanje, Ronald Mbewe, Sunshine Mfungwe, Zakaliah Mphande, Hannock Tweya. **Mozambique:** Idiovino Rafael. **South Africa:** Patti Apolles, Eunice Beneke, Siphephelo Dlamini, Claire Edson, Brian Eley, Jonathan Euvrard, Geoffrey Fatti, Bridgette Goeieman, Ashraf Grimwood, David Huang, Susan Hugo, Zahiera Ismail, Lauren Jennings, Thulile Mathenjwa, Lizette Monteith, Zamuxolo Mshweshwe, Mfundi Ntuli, EN Ndlovu, Hloniphile Ndlozi, Sylvia Noyakaza, Hans Prozesky, Helena Rabie, Nosisa Sipambo, Karl-Günter Technau, Thokozani Tembe, Nontando Xaba**. Zambia:** Thandiwe Njobvu, Mary Munthaly, Elly Mwetwa, Gillian Kabeba, Derrick Mwendafilumba, Ethel Maanguka, Nelly Manyika, Chalwe Mwansa, Future Banda, Dickson Mwenda, Abel Bwalya, Leah Shapi, Kasapo Syame, Rita Sashi, Chisha Mulenga, Ruth Nanyangwe. **Zimbabwe:** Cleophas Chimbetete, A. Chinofunga, J. Mhike, E. Mubvigwi, F. Nyika, Kumbirai Pise Quarter.

**IeDEA West Africa**

Shino Chassagne Arikawa, Renaud Becquet, Charlotte Bernard, François Dabis, Sophie Desmonde, Désiré Dahourou, Didier Koumavi Ekouevi, Antoine Jaquet, Julie Jesson, Valeriane Leroy, Karen Malateste, Elodie Rabourdin, Thierry Tiendrebeogo.

**Benin:** Michée Assogba, Marcelline d'Almeida, Djimon Marcel Zannou , Ghislaine Hounhoui. **Burkina Faso:** Denise Bere, Armel Poda, Gbolo Pooda, Richard Traore. **Côte d'Ivoire:** Yao Abauble, Ouattara Abby, Patrick Acquah, Valérie Andoble, Yobo N'Dzama Aude, Jean-Claude Azani, Oka Berete, Jacques Daple Beugre, Caroline Yao Bohoussou, Simon Boni Emmanuel Brou, Henri Chenal, Abdoulaye Cissé, Nambate Coulibaly, Marie Evelyne Dainguy, Marcelle Daligou, Toni Thomas d'Aquin, Claude Desire Dasse, Madeleine Amorissani Folquet, Guy Gnepa, Olivier Gobe, Salif Guira, Denise Hawerlander, Apollinaire Horo, Guillaume Kanga, Zobo Konan Eugène Messou, Kla Albert Minga, Raoul Moh, Marie Sylvie N'Gbeche, Patricia Ogbo, Mathieu Oulai, SE Stéphanie, Tanoh Eboua, Itchy Max Valère. **Ghana:** Adwoa Kumiwa Asare Afrane, Esther Akrofi, John Christian Andoh, Lorna Renner. **Mali:** Awa Bagayoko, Kadidiatou Bagayoko, Abdou Salam Bah, Alima Berthe, Boureïma Coulibaly, Fatimata Coulibaly, Yacouba Aba Coulibaly, Aïssata Diakité, Fatoumata Bocoum, Fatoumata Boré, Fatoumata Dicko, Odile Koné, Mariam Sylla, Assitan Tangara, Mamadou Traoré. **Senegal:** Moussa Seydi. **Togo:** Edmond Amegatse, Julienne Djossou, Elom Takassi, Sénam Palanga.
